# Diosbulbin B-Induced Mitochondria-Dependent Apoptosis in L-02 Hepatocytes is Regulated by Reactive Oxygen Species-Mediated Autophagy

**DOI:** 10.3389/fphar.2019.00676

**Published:** 2019-06-19

**Authors:** Jing Ye, Mei Xue, Yamin Liu, Sirui Zhu, Yu Li, Xiaoli Liu, Danhong Cai, Jia Rui, Liang Zhang

**Affiliations:** ^1^College of Pharmacy, Nanjing University of Chinese Medicine, Nanjing, China; ^2^College of Preclinical Medicine, Nanjing University of Chinese Medicine, Nanjing, China; ^3^Department of Pharmacy, Zhongda Hospital, Southeast University, Nanjing, China

**Keywords:** diosbulbin B, autophagy, mitochondrial dysfunction, apoptosis, hepatocytes

## Abstract

**Aim:** Diosbulbin B (DB) is a major diterpenoid compound found in *Dioscorea bulbifera* L, a traditional medicinal herb in China. Clinical reports have confirmed that *Dioscorea bulbifera* L. can induce significant hepatotoxicity. In this study, we showed that DB can induce mitochondria-dependent apoptosis and investigated the role of autophagy in DB-induced hepatotoxicity in L-02 hepatocytes.

**Methods:** L-02 hepatocytes were treated with different concentrations of DB for 48 h, after which indicators of autophagy and apoptosis were measured. 3-Methyladenine (3-MA) and rapamycin (Rapa) were used as inhibitor and agonist of autophagy, respectively. Furthermore, the reactive oxygen species (ROS) scavenger N-acetyl-l-cysteine (NAC) was used in combination with DB to evaluate the relationship between ROS and autophagy.

**Results:** L-02 cell viability was significantly decreased after treatment with DB for 48 h. Additionally, DB induced concentration-dependent apoptosis and autophagy and increased the activities of caspase-3, caspase-9, alanine aminotransferase (ALT), and aspartate transaminase (AST), and induced excessive leakage of lactate dehydrogenase (LDH). Inhibition of autophagy by 3-MA increased DB-induced apoptosis, resulting in aggravation of hepatotoxicity. Conversely, treatment with Rapa increased malondialdehyde (MDA) content and reduced superoxide dismutase (SOD) activity. Moreover, we found that DB treatment increased the level of intracellular ROS, decreased the mitochondrial membrane potential (MMP) and adenosine triphosphate (ATP) production, and caused abnormal opening of the mitochondrial permeability transition pore (mPTP), which were finally restored by the ROS scavenger NAC.

**Conclusions:** Accumulation of ROS can induce mitochondria-dependent apoptosis and likely to play a key role in DB-induced hepatocellular injury. Activation of autophagy may inhibit apoptosis, but also reduces antioxidant capacity.

## Introduction

Diosbulbin B (DB) is a diterpene lactone compound in *Dioscorea bulbifera* L., which is well known for its unique therapeutic effect on treating thyroid disease in China (Wang et al., 2012). However, clinical studies show that *Dioscorea bulbifera* L. can induce significant hepatotoxicity after long-term oral administration (Huang et al., 2013). Furthermore, recent research identified DB as a major toxic ingredient (Niu et al., 2016). A previous report showed that DB induced oxidative stress and cholestasis in animal experiments (Ma et al., 2014). According to its metabolic characteristics, the furan moiety of one DB is responsible for the formation of electrophilic species that eventually lead to liver damage (Lin et al., 2016). The mitochondrion is an essential organelle that generates energy and maintains redox homeostasis in cells. Mitochondrial dysfunction can therefore inhibit the chain of energy supplementation and cause oxidative stress, as well as a shift in metabolic pathways (Halbrook et al., 2018). Further, the breakdown of inner mitochondrial transmembrane potential (MMP) is an early stage of mitochondria-dependent apoptosis (Wang et al., 2015), whereby excessive production of reactive oxygen species (ROS) from the mitochondrial electron transport chain (ETC) attacks lipids and proteins on the membrane. The mutation, deletion, or insertion of mitochondrial DNA (mtDNA) caused by ROS ultimately creates an irreversible cycle that triggers mitochondrial dysfunction (Vakifahmetoglunorberg et al., 2017).

Autophagy is a self-digestive mechanism that serves to remove damaged organelles. A recent research found that accumulation of ROS can induce the generation of autophagosomes (Li et al., 2015). However, autophagy under stress conditions induces adverse cellular effects. Autophagy contributes to reduction in the level of ROS by degrading oxidized proteins (Ureshino et al., 2014). However, excessive autophagy induces pathways that facilitate non-apoptotic cell death, which is also called autophagic death (Robert et al., 2015). It has been shown that ROS can initiate autophagic death during enhanced oxidative stress injury. Consequently, autophagy lays a critical and intricate role in the cellular self-regulation process. Previous studies have demonstrated that DB can induce hepatocyte apoptosis (Niu et al., 2014) and mitochondrial damage (Shang et al., 2007), along with the presence of oxidative stress (Ma et al., 2014) in animal experiments. However, the role of autophagy in DB-induced hepatotoxicity and its effects on cell injury have not been characterized.

In this study, we observed mitochondrial dysfunction and activation of autophagy in DB-treated L-02 hepatocytes, evaluated the mechanisms of DB-induced hepatotoxicity, and evaluated the effects of autophagy in cell injury.

## Method and Materials

### Materials and Reagents

**Figure 1:**
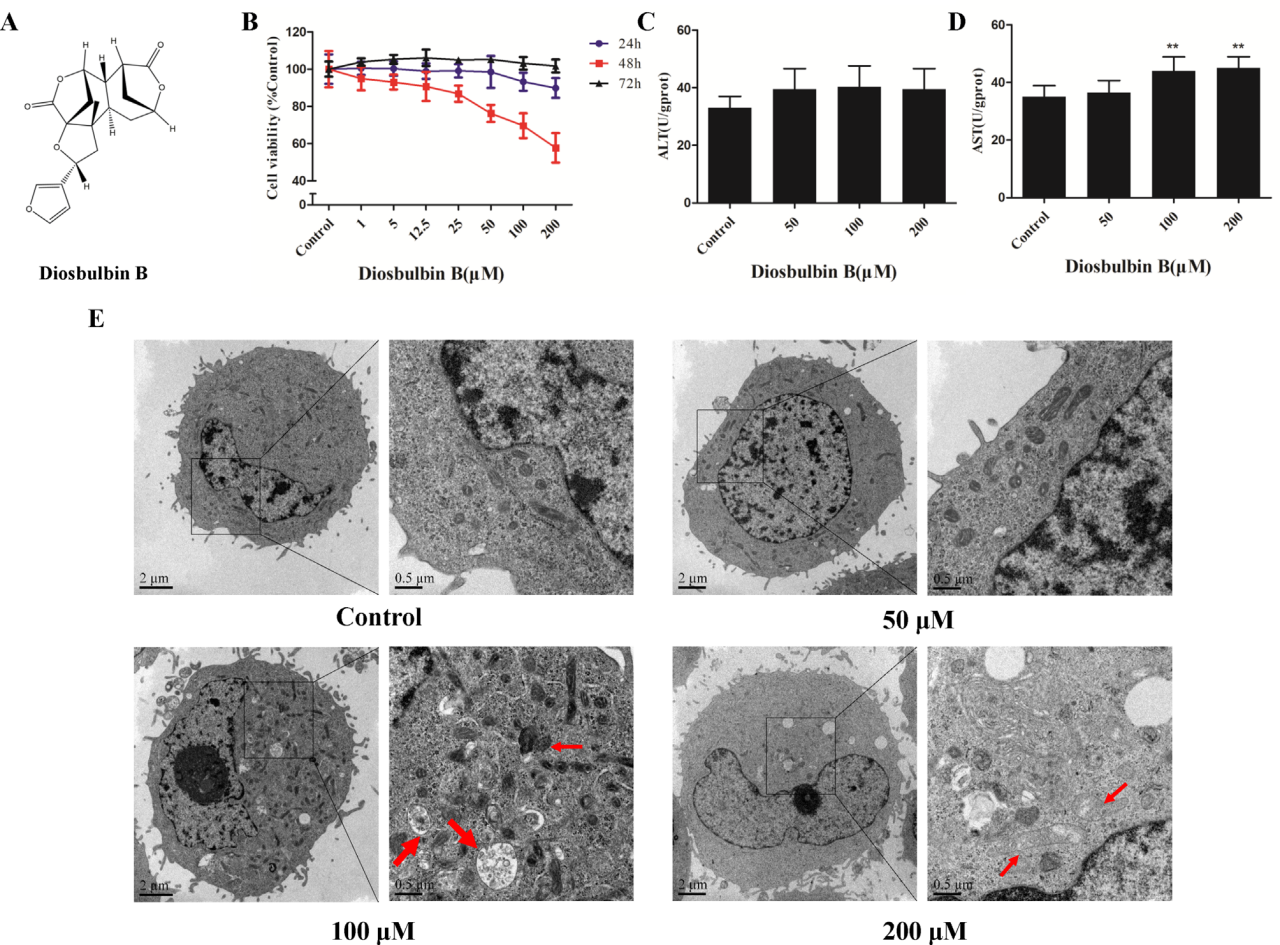
Diosbulbin B (DB) induced cell injury in a dose-dependent manner. **(A)** Chemical structure of DB. **(B)** Vitality of L-02 cells after the treatment with DB at different concentrations for 24, 48, and 72 h. **(C, D)** Alanine aminotransferase (ALT) and aspartate aminotransferase (AST) activity in response to treatment with different concentrations of DB (0, 50, 100, and 200 µM) for 48 h were measured. Results were calculated from one of three independent experiments (n = 3). **P < 0.01, compared with the 0-µM DB group (control group). **(E)** Microscopic observation of cells treated with DB (0, 50, 100, and 200 µM) for 48 h. Thick arrow autophagosome structure; Thin arrow, damaged mitochondria.

DB, purity ≥98% (**Figure 1A**), was purchased from Solarbio Life Sciences Co, Ltd (Beijing, China), and characterized by high-performance liquid chromatography (HPLC). N-acetyl-l-cysteine (NAC), 3-methyladenine (3-MA), and rapamycin (Rapa) were obtained from Apexbio. 3-(4,5-Dimethyl-2-thiazolyl)-2,5-diphenyl-2-H-tetrazolium bromide (MTT) was obtained from Sigma. Monodansylcadaverine (MDC) was obtained from Nanjing KeyGEN Biotech Co, Ltd. Kits for detecting alanine aminotransferase (ALT), aspartate aminotransferase (AST), malondialdehyde (MDA), superoxide dismutase (SOD), and adenosine triphosphate (ATP) were obtained from Nanjing Jiancheng Co, Ltd. A kit for detection of lactate dehydrogenase (LDH) was purchased from Maibo Biotechnology Co., Ltd. Other diagnostic kits were purchased from Beyotime Biotechnology. The AnnexinV Alexa Fluor647/PI apoptosis-detecting kit was purchased from Fcmacs Biotech Co., Ltd. Anti-body to GAPDH was from Abways Technology. Anti-bodies to Bax, Bcl-2, p62, caspase-3, LC3, Beclin-1, and goat anti-rabbit IgG were purchased from Proteintech Group, Inc. The kit for detecting mitochondrial permeability transition pores (mPTP) was purchased from BestBio Co., Ltd Science.

### Cells and Culture Conditions

The human liver cell line (L-02) was obtained from the China Center for Type Culture Collection at Wuhan University. All cells were cultured in DMEM containing 10% fatal bovine serum (FBS) (v/v), 100 U/ml penicillin, and 100 μg/ml streptomycin at 37°C in 5% CO_2_.

### Measurements of Cell Viability

Cell viability was measured using 3-(4,5-dimethylthiazol-2yl-)-2,5-diphenyl tetrazolium bromide (MTT) assay. In general, cells growing in the exponential growth phase were seeded in 96-well plates at 1×10^4^ cells/ml. After incubation with various concentrations of DB for 24, 48, and 72 h, 200 µl of 5 mg/ml MTT was added and the cells were incubated for 4 h at 37°C. After removal of the incubation medium and formazan was dissolved in 150 µl DMSO for 15 min at 37°C, the absorbance was read at 490 nm using a microplate reader (TECAN Infinite M200 PRO).

### Assessment of Biological Indicators

L-02 cells were seeded in 6-well-plates at a density of 6×10^5^ cells/well, and incubated with different concentrations of DB for 48 h. The cells were divided into different groups for further investigation as indicated in each experiment. Supernatants were collected to detect the levels of AST, ALT, and LDH.

After ultrasonic disruption on ice, the cell lysates were used for the detection of ATP, MDA, and SOD.

### Electron Microscopy

L-02 cells were harvested and washed with PBS. The cells were fixed in 2.5% glutaraldehyde for 2 h. After rinsing in 0.1 M sodium cacodylate three times, the cells were incubated in 2% osmiumtetroxide for 2 to 3 h and dehydrated using an ethanol and acetone gradient steps at 4°C for 15 to 20 min. After fixation, 50 to 60 nm sections were cut using an ultramicrotome. The sections were then stained with 3% uranyl acetate and lead citrate, and observed under transmission electron microscope.

### Determination of Reactive Oxygen Species Production

Intracellular ROS levels in L-02 cells were assessed in six-well plates using 2,7-dichlorofluorescein diacetate (DCFH-DA). The cells were washed twice with PBS and incubated with 1 μM DCFH-DA in the dark for 30 min at 37°C. The plates were then analyzed using a fluorescence microplate reader with the excitation and emission wavelengths of 488 and 525 nm, respectively.

### Detection of MMP (ΔΨm)

The ΔΨm of L-02 cells was analyzed according to the manufacturer’s protocol (Beyotime Institute of Biotechnology Nanjing, China). 5,5′,6,6′-Tetra-chloro-1,1′,3,3′-tetraethylbenzimidazolyl-carbocyanine iodide (JC-1) fluorescent dye was incubated with cells at 37°C for 20 min. The cells were then washed with incubation buffer for three times, and the change of fluorescence was observed with a fluorescent microscope under the excitation wavelengths of 488 and 525 nm (ZEISS inverted fluorescence microscope).

### Flow Cytometry

Annexin V-Alexa Flour 647/PI double staining assay was used to evaluate the apoptosis (Fcmacs Biotech Co., Ltd). Cells were harvested after the incubation period and rinsed twice with PBS, then resuspended in binding buffer. According to the manufacturer’s protocol, 5 μl Annexin V/Alexa Fluor 647 and 10 μl, 20 μg/ml PI staining dyes were added to cells at a concentration of 1×10^6^ cells/ml. After incubation at room temperature in the dark for 15 min, cells were detected with flow cytometry under the excitation wavelengths of 488 and 635 nm (BD Biosciences AccuriC6).

### Caspase-3 and Caspase-9 Activity Assay

The activities of caspase-3 and caspase-9 were measured using a commercially available kit (Beyotime Institute of Biotechnology, Nanjing, China) according to the manufacturer’s protocol. Cells were homogenized in lysis buffer for 30 min on ice and centrifuged at 4°C at 12,000 rpm for 15 min. Then, 10 μl of Ac-DEVD-pNA or Ac-LEHD-pNA was added, and incubated at 37°C for 24 h, after which absorbance was detected at 405 nm using a microplate reader.

### MDC Staining

An MDC staining assay was used to detect the presence of autophagic vacuoles. Cells were seeded in 24-well plates at a density of 1.5 × 10^4^ cells/ml. After treatment with DB for 48 h, cells were incubated with MDC staining dye for 30 min and washed three times with wash buffer. Autophagic vacuoles were then observed with a fluorescent microscope under the excitation wavelength of 326 nm.

### Analysis of Mitochondrial Permeability Transition Pore

Evaluation of mPTP was performed according to the manufacturer’s protocol. Cells were collected at a density of 1×10^6^ cells/ml and suspended in PBS. Then, 5-µl BBcellProbeTMM61 staining probe and 5 µl of Quenching agent were added. After incubation in 37°C for 15 min, cells were centrifuged at 2,000 r/min for 5 min, then detected with flow cytometry under the excitation wavelength of 488 nm (Becton-Dickinson FACS Calibur) and analyzed using BD CellQuest pro.

### Immunoblotting

Hepatocytes were seeded in six-well plates at a density of 3×10^5^ cells/ml and allowed to adhere for 12 h. The cells were then treated with medium containing various concentrations of DB for 48 h cells were lysed in RIPA buffer to obtain total protein, after which a BCA protein assay was applied to determine the concentration. After degeneration, proteins were separated by 10% or 12% SDS-polyacrylamide gel electrophoresis and transferred to nitrocellulose membranes. The membranes were then blocked with 5% skim milk and reacted overnight with antibodies specific to Bcl-2, Bax, Beclin-1, p62, LC3, and GAPDH at 4°C. The immunoblots were incubated with anti-rabbit secondary antibody and visualized by chemiluminescence detection.

## Statistical Analysis

GraphPad Prism 5 software was used for statistical analysis, and results were expressed as mean ± standard deviation (SD). Analysis of variance (ANOVA) was used for comparison among more than two groups; *P* < 0.05 was considered statistically significant.

## Results

### Diosbulbin B Induced Cytotoxicity in L-02 Cells

The viability of L-02 cells decreased significantly after treatment with up to 200 μΜ DB at 48 h in a dose-dependent manner (**Figure 1B**). The cells were incubated in solvent (1‰ DMSO) or DB (50, 100, and 200 μM) for 48 h; then, a sequence of diagnostic tools was used to analyze. As shown in **Figure 1C and D**, the levels of AST and ALT increased after treatment with DB. DB treatment also resulted in increased cellular MDA content and reduced SOD activity (**Supplementary Figure S1**).

### Diosbulbin B Induced Autophagy and Apoptosis in L-02 Hepatocytes

**Figure 2:**
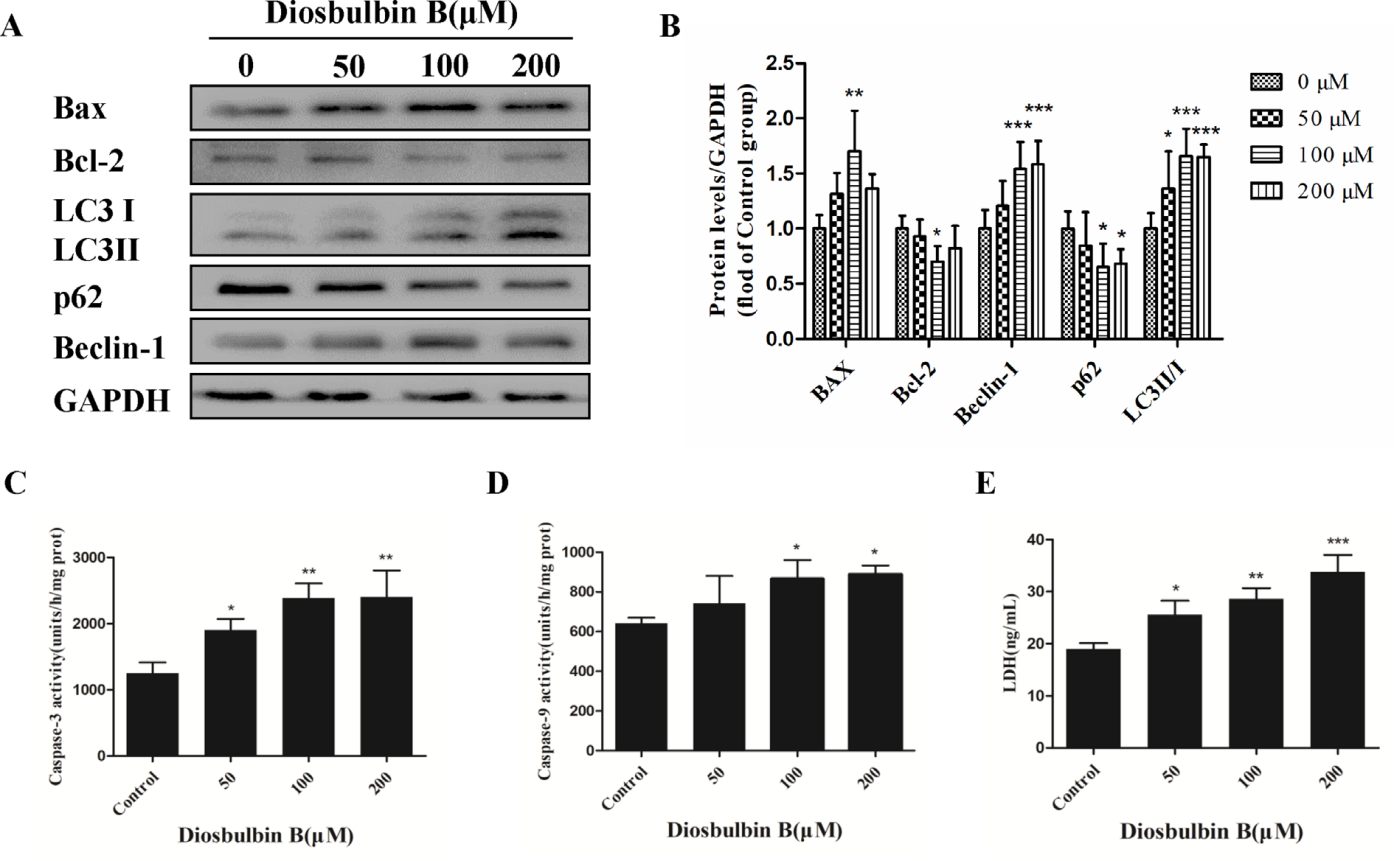
Increased autophagy and apoptosis in L-02 cells after the treatment with DB (100 µM). **(A, B)** DB affected the expression of autophagy and apoptosis related proteins in L-02 cells at 48 h. **(C, D)** Activities of caspase-3 and caspase-9 were measured using a fluorescence microplate reader. The results are presented as mean ± SD (n = 3). **(E)** Leakage of LDH was analyzed with detection kits. The results are presented as mean ± SD (n = 3). ***P < 0.001, **P < 0.01, and *P < 0.05 compared to control group.

The microstructures of hepatocytes in **Figure 1E** showed the structure of autophagosomes and autolysosome following treatment with DB (50, 100, and 200 µM). As shown in **Figure 2A and B**, the expressions of LC3 II/I and Beclin-1 increased in a dose-dependent manner after 48 h of treatment with DB, while the expression of p62 decreased. MDC staining results demonstrated enhanced fluorescence signals in the DB treatment groups (**Supplementary Figure S2**). These results suggested the activation of autophagy during DB-induced hepatocyte injury. In addition, enhanced protein expression of Bax, up-regulated activities of caspase-3 and caspase-9, and excessive leakage of LDH revealed that DB can induce cell apoptosis in L-02 hepatocytes (**Figure 2C-E**).

### Autophagy Protected Against Diosbulbin B-Induced Cell Apoptosis, but Reduced Antioxidant Capacity

**Figure 3:**
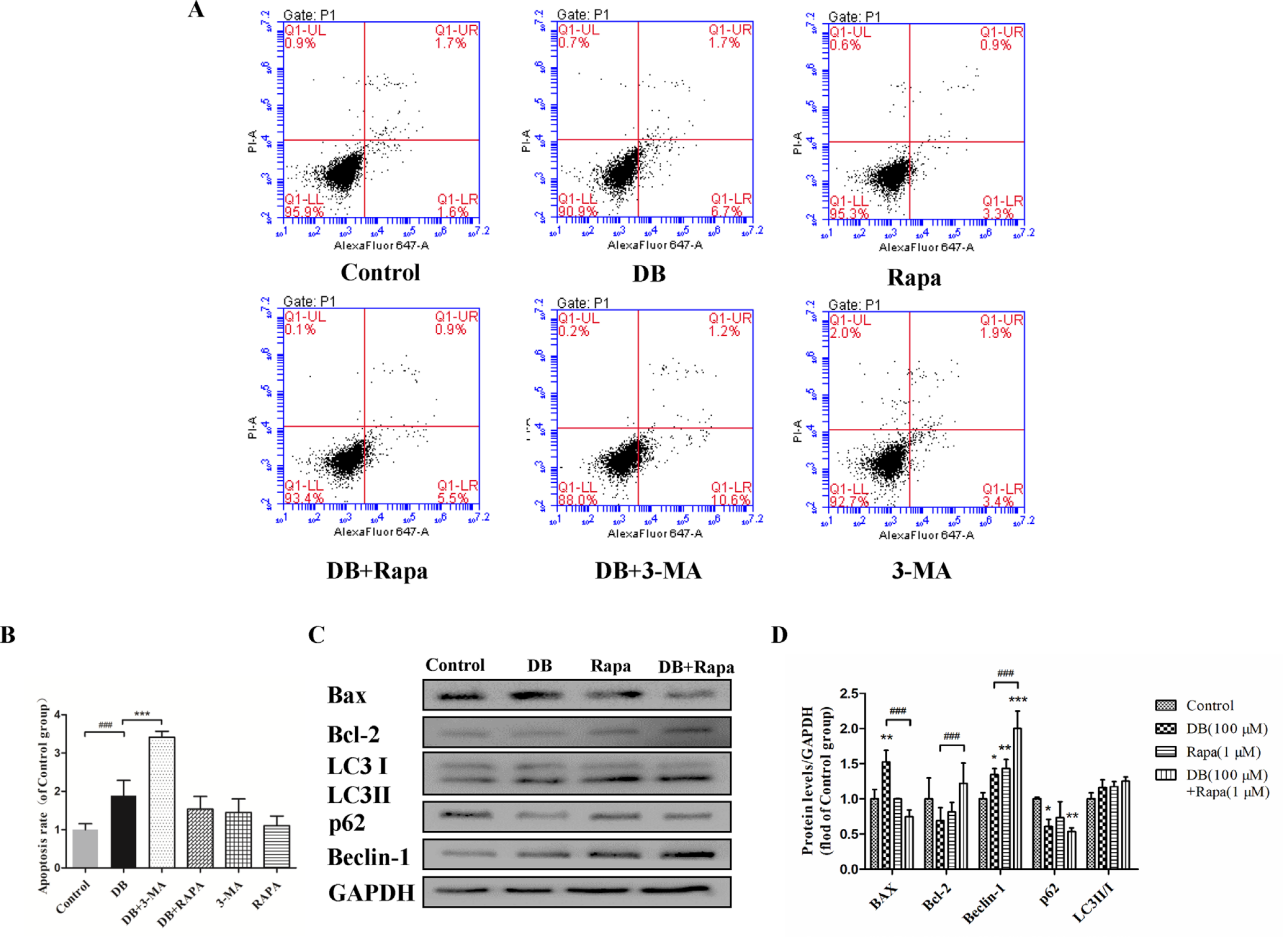
Activation of autophagy regulated DB-induced apoptosis in L-02 hepatocytes. Rapa (1 µM) and 3-MA (2.5 mM) were used as an agonist and an inhibitor of autophagy, respectively. Cells were divided into six groups, control group treated with 1‰ DMSO, Rapa (1 µM), 3-MA (2.5 mM), 100 µM DB only, and co-treatment of DB with Rapa or 3-MA. **(A, B)** Apoptosis was assessed using flow cytometry. **(C, D)** The levels of autophagy and apoptosis related proteins after 48-h treatment were measured by Immunoblotting. The results are presented as mean ± SD (n = 3) and calculated from at least three independent experiments. ***P < 0.001, compared to control group. ^###^ *P* < 0.001, compared to DB group.

**Figure 4:**
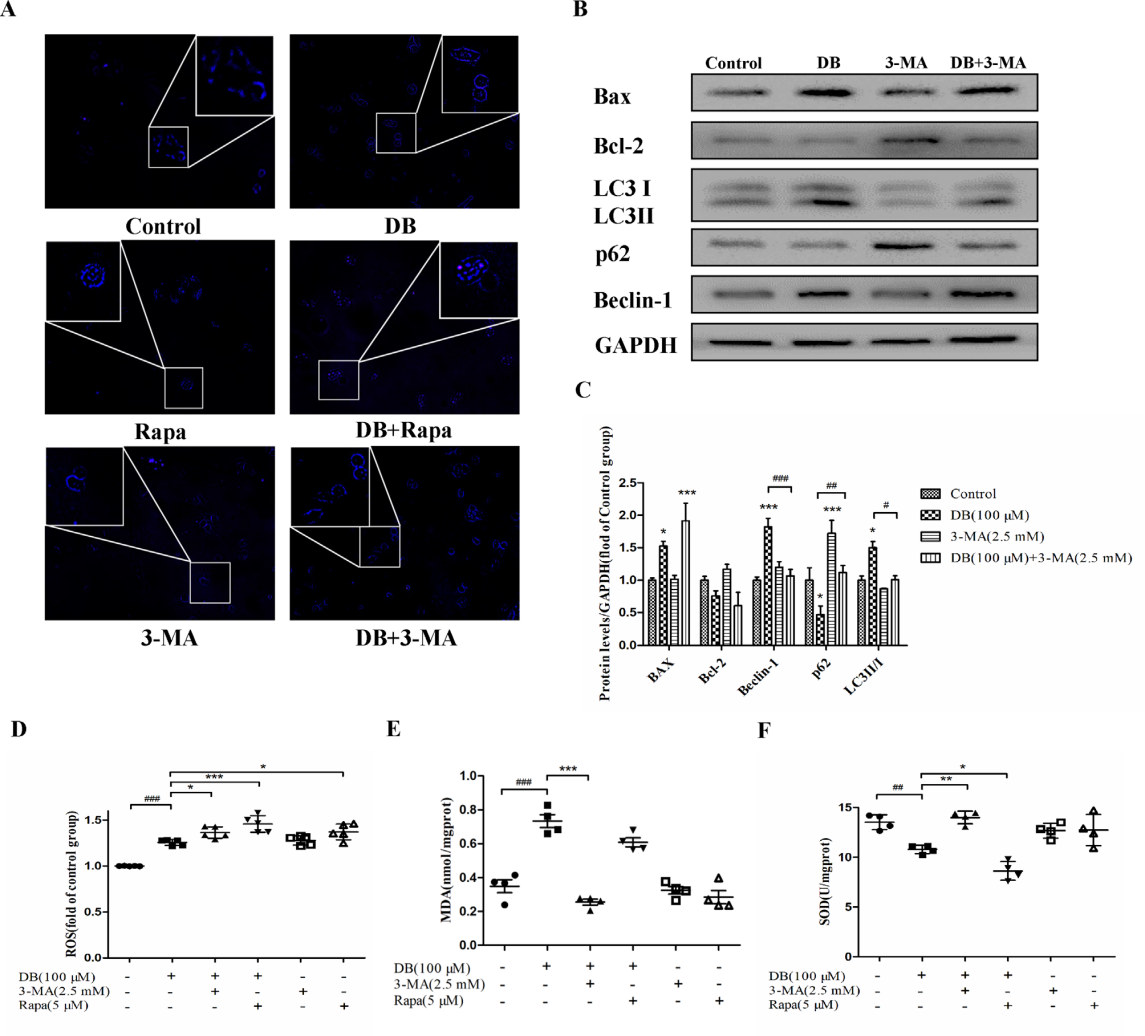
Activation of autophagy regulated antioxidant capacity. Cells were divided into six groups and treated for 48 h as described in the **Figure 3** caption. **(A)** Monodansylcadaverine (MDC) staining assay was applied to detect autophagy. **(B, C)** The expression of autophagy and apoptosis related proteins in the different groups. **(D)** The fluorescence intensity of ROS. **(E, F)** The levels of MDA and SOD were analyzed using a microplate reader. The results are shown as mean ± SD (n = 3) and calculated from at least three independent experiments. ***P < 0.001, **P < 0.01, *P < 0.05, compared to the control group. ^###^ *P* < 0.001, compared to the DB group.

To explore the role of autophagy in DB-induced cell apoptosis, 3-MA (2.5 mM) (Wang et al., 2014) and Rapa (1 μM) (Fu et al., 2016) were used as inhibitor and agonist of autophagy. As shown in **Figure 4A**, enhanced fluorescence intensity was observed in Rapa-treated group, indicating more autophagic vacuoles in L-02 cells. The results of accelerated Annexin V-Alexa Flour647/PI staining showed that inhibition of autophagy in the 100-µM DB group cell apoptosis, and Rapa had the opposite effect (**Figure 3A and B**). Immunoblotting results demonstrated that the expression of apoptosis related proteins Bax/Bcl-2 was in accordance with the results obtained using flow cytometry (**Figures 3C, D** and **4B, C**). However, we observed increased MDA level and decreased SOD activity in the Rapa recombination group, and adverse results were found in 3-MA groups (**Figure 4E and F**). Additionally, accumulation of ROS was not affected in either the 3-MA or Rapa groups (**Figure 4D**).

### Diosbulbin B Caused Mitochondrial Dysfunction

After treatment with DB for 48 h, the ultrastructure of hepatocytes exhibited intracellular vacuolation and mitochondrial injury (**Figure 1E**). To confirm mitochondrial dysfunction, the MMP was evaluated using JC-1 staining. The results showed that cells incubated with DB displayed bright green fluorescence, whereas untreated cells exhibited red, representing a decrease in membrane potential (**Supplementary Figure S3**). Increased level of ROS further demonstrated abnormal function of mitochondria (**Supplementary Figure S4**).

### Removal of Reactive Oxygen Species Mitigated Mitochondrial Dysfunction and Decreases Autophagy

**Figure 5:**
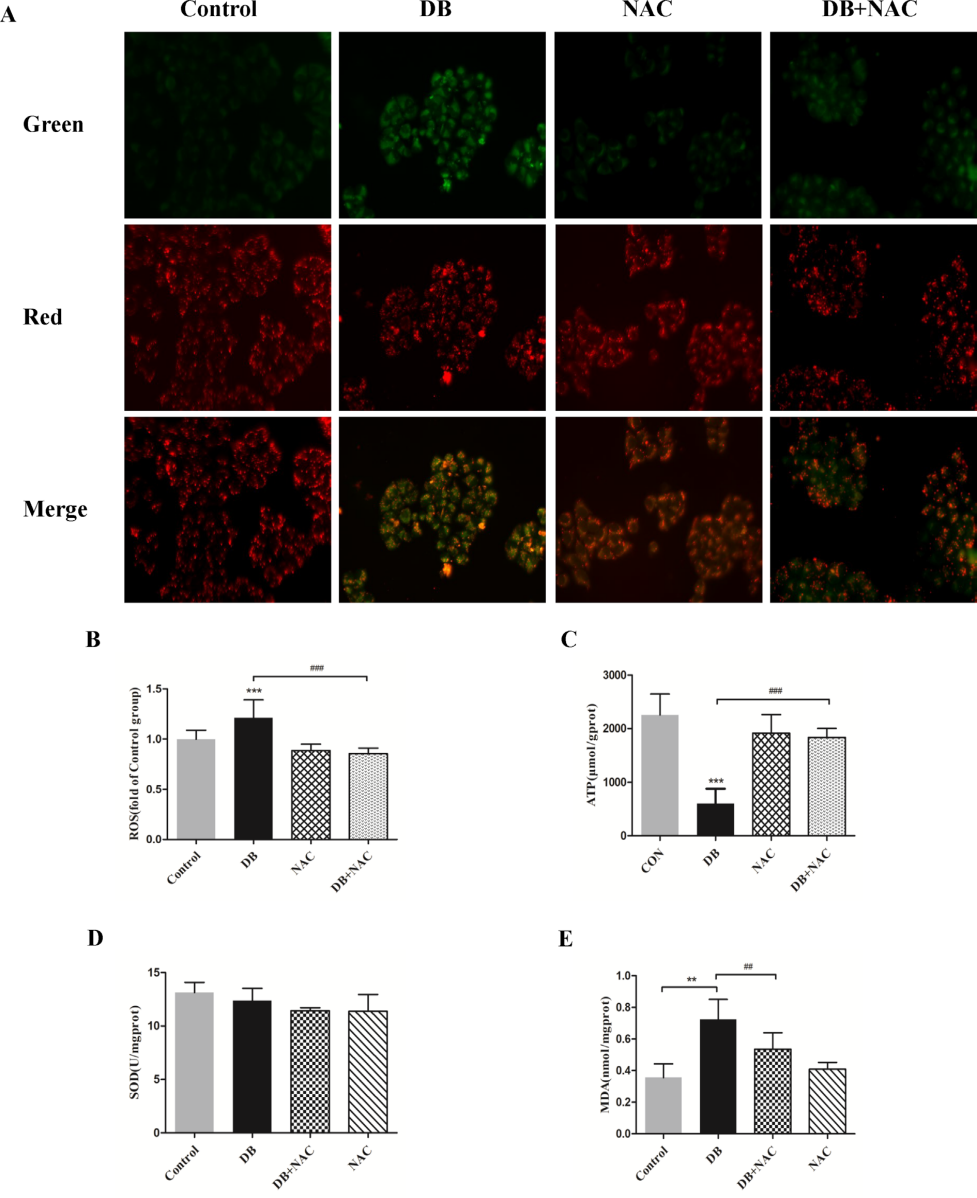
Clearance of radical mitigated cell damage in DB-treated groups. Cells were divided into a control group, DB treatment group, N-acetyl-l-cysteine (NAC, 5 µM) treatment group, or DB and NAC cotreatment group. **(A)** Mitochondrial membrane potential was analyzed using JC-1 staining. **(B)** The fluorescence intensity of ROS. **(C)** ATP levels in each of the treated groups. **(D, E)** Levels of SOD and MDA were analyzed using a microplate reader. The results are presented as mean ± SD (n = 3) and calculated from at least three independent experiments. ***P < 0.001, **P < 0.01, compared to the control group. ^###^ *P* < 0.001, ^##^ *P* < 0.01, compared to the DB group.

**Figure 6:**
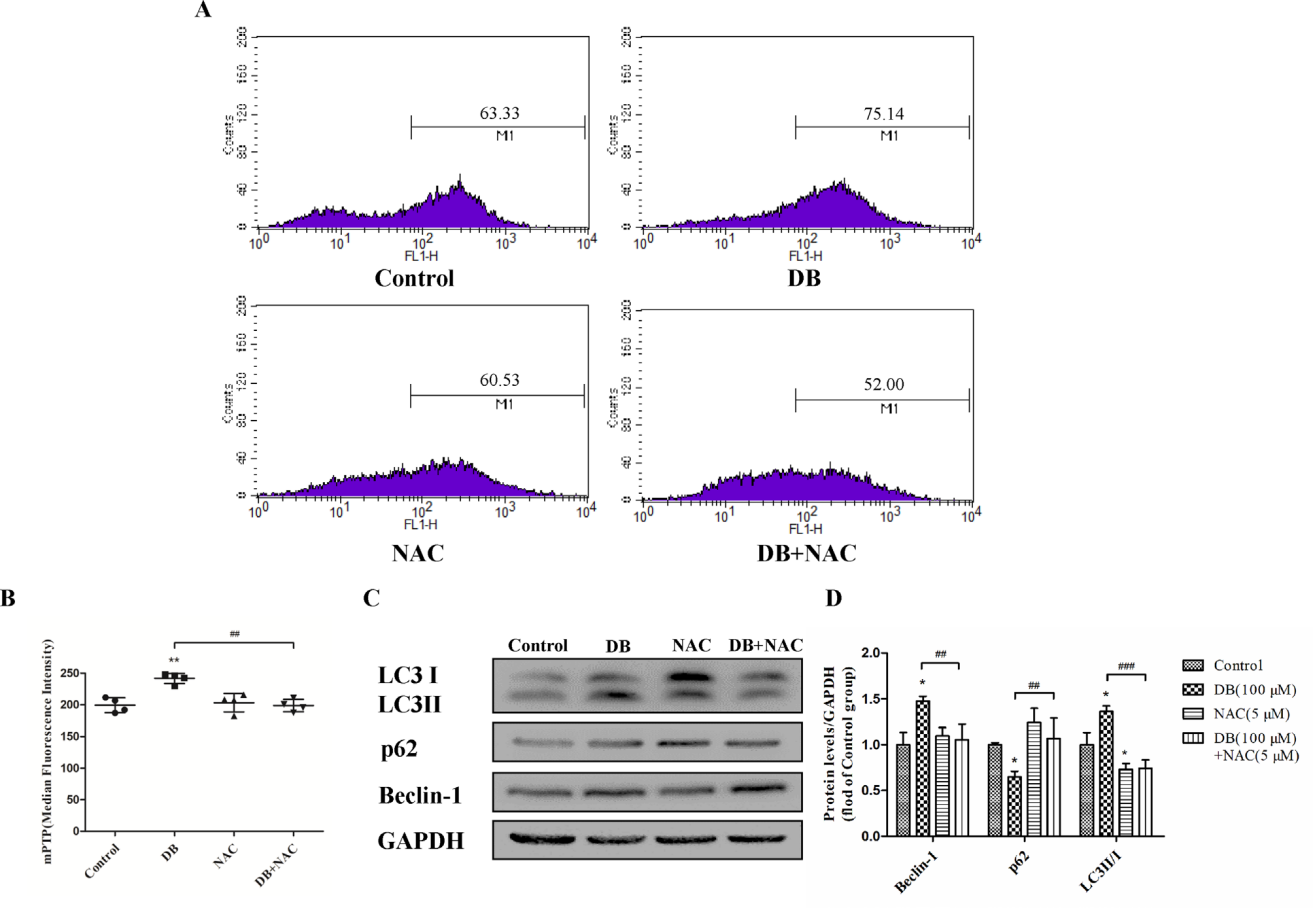
NAC reduced the opening of the mitochondrial permeability transition pore (mPTP). **(A, B)** mPTP opening was detected using flow cytometry. **(C, D)** The expression of autophagy related proteins in the different groups. The results are shown as mean ± SD (n = 3) and calculated from at least three independent experiments. **P < 0.01, *P < 0.05, compared to the control group. ^###^ *P* < 0.001,^##^ *P* < 0.01, compared to the DB group.

NAC (5 mM) (Ying et al., 2014) was used as a ROS scavenger to eliminate the over-production of ROS induced by DB (100 µM) (**Figure 5B**). Improved cell viability demonstrated that ROS is, potentially, a key factor in DB-induced cell death (**Supplementary Figure S5**). The results in **Figure 5A and C** showed that NAC restored the MMP and increased production of ATP, but showed no significant effect on the activity of SOD (**Figure 5D**). The abnormal opening of mPTP was restricted after the treatment with NAC (**Figure 6A and B**), and the level of MDA was reduced (**Figure 5E**). Reduced expression of LC3 II/LC3 I and Beclin-1 revealed decreased autophagy (**Figure 6C and D**).

## Discussion

Mitochondria are pivotal organelles, generally considered as “power plants” of cells. They generate energy during cell differentiation, protein synthesis, and catabolism, and also participate in immune responses and other biological processes (Yamashita and Kanki, 2017). As such, mitochondrial dysfunction can cause extensive cellular dysfunction, which can trigger autophagy (Wible and Bratton, 2018), apoptosis (Meng et al., 2016), and other forms of cellular injury. Autophagy is a cellular recycling process that facilitates cell survival and modulates the process of apoptosis and inflammation (Rabinowitz and White, 2010). However, growing evidence suggests that unrestrained autophagy can trigger apoptosis by interrupting the balance between protein degradation and synthesis (Klionsky and Emr, 2000; Kroemer and Levine, 2008). The present study demonstrates that DB can induce mitochondria-dependent cell apoptosis in L-02 cells that is regulated by ROS-mediated activation of autophagy.

Autophagy is a dynamic process that consists a series of membrane structural changes initiated by the formation of autophagosomes (Meng et al., 2016). The autophagosome features a double-membraned vesicle, and resembles structures observed in our study (**Figure 1E**). The results of this study showed that DB facilitated the formation of autophagosomes in L-02 cells in a dose-dependent manner. The autophagy protein microtubule-associated protein 1 light-chain 3 (LC3) exists in two forms during activation of autophagy (Fu et al., 2016). After LC3 is cleaved to LC3 I by Atg4 in the cytoplasm, phosphatidylethanolamine (PE) and LC3 I form a LC3-II-PE complex, which participates in autophagic membrane extension and is distributed on the membranes of autophagic vacuoles (Yun et al., 2018). Integration of the LC3-II-PE complex and SQSTM1(p62) transfers the wasted substance, which is degraded by autolysosomes, into autophagosomes (Katsuragi et al., 2015). As such, the contrasting expression of LC3 II/I and p62 is an enhanced signal of autophagic flux. In our study, the ratio of LC3 II/I protein expression increased and the level of p62 was reduced in response to DB treatment. We further evaluated the expression of Beclin-1, which is a platform molecule involved in activation of autophagy (Salminen et al., 1900). The enhanced expression of Beclin-1 in the DB-treated groups in our study demonstrated that DB can induce activation of autophagy (**Figure 2A and B**). MDC staining indicated similar results (**Supplementary Figure S2**). Increased leakage of LDH revealed that DB treatment induced cell membrane damage (**Figure 2E**) (Yang et al., 2018). Caspases involved in apoptosis can be divided into two groups: initiators and executioners (Guo et al., 2015). As an initiator, caspase-9 can activate caspase-3 and trigger the apoptotic cascade (Adamczyk et al., 2010). In addition, previous studies have demonstrated that activation of caspase-3 and caspase-9 is related to mitochondrial dysfunction (Guo et al., 2018).

As shown in **Figure 2C and D**, the activities of caspase-3 and caspase-9 suggested the presence of apoptosis, as did the up-regulation of Bax/Bcl-2 level. To determine the relationship between autophagy and apoptosis in DB-induced hepatocyte injury, we used 3-MA and Rapa as inhibitor and agonist, respectively, of autophagy. Treatment with 3-MA exacerbated DB-induced apoptosis and Rapa treatment decreased DB-induced apoptosis (**Figure 3A and B**). Increased expression of Bax/Bcl-2 in the 3-MA group also demonstrated that inhibition of autophagy deteriorated DB-induced apoptosis (**Figure 3C and D**). These results supported that activation of autophagy induced by DB hinders apoptosis.

TEM results showed alterations in mitochondrial structure in response to high concentrations of DB (**Figure 1E**). For further verification of mitochondrial dysfunction, JC-1 staining assay was used to measure MMP, which is an indicator of mitochondrial metabolism and vitality (Carvalho et al., 2017). The results showed the depolarization of MMP as shown in **Supplementary Figure S3**. The combination of Bax and Bcl-2 enhances the permeability of the mitochondrial membrane, promoting the release of internal proteins into cytoplasm (Wang et al., 2014). Previous studies have shown that mitochondrial dysfunction facilitates excessive production of ROS, leading failure of energy supplementation and cell death (Go et al., 2015). Excessive ROS results in oxidative stress and damages mtDNA which damages the ETC (Zorov et al., 2014). Our results indicated that DB promoted the production of ROS (**Supplementary Figure S4**), suggesting a relationship between oxidative stress and mitochondrial dysfunction.

Accumulation of ROS contributes to the oxidation reaction and causes extended opening of the mitochondrial membrane permeability transition pore and ATP-sensitive channels, which results in cell apoptosis (Wu et al., 2018). Correspondingly, abnormal opening of the mitochondrial membrane channel was observed in this study (**Figure 6A and B**). As noted, autophagy is a protective pathway that maintains homeostasis in cells (Ryter et al., 2014). Activation of autophagy degrades oxidized mitochondria and removes sources of ROS (Ye et al., 2017). However, ROS-mediated activation of autophagy in cells potentially regulates cell survival in a double-edged way. As ROS can trigger selective “pexophagy,” which causes degradation of antioxidant enzymes (Wible and Bratton, 2018). Interestingly, we found that the levels of ROS increased after incubation with either 3-MA or Rapa. Additionally, enhanced antioxidant capacity was observed in the 3-MA group, with increased level of SOD and a reduction in MDA level (**Figure 4E and F**). Based on these results, we hypothesized that excessive activation of autophagy caused degradation of antioxidant enzymes. We further investigate whether elimination of ROS affected mitochondrial function, using NAC as a ROS scavenger (**Figure 5B**). Removal of ROS increased intracellular ATP production and prevented excessive openness of the mPTP. In addition, decreased expression of autophagy-related proteins was observed in cells treated with NAC (**Figure 6C and D**). As such, these data demonstrated that ROS production is primary cause of DB-induced mitochondrial damage, and that autophagy was a protective measure for cells to prevent further damage. However, ROS-induced autophagy in cells treated with DB had the negative consequence of excessive clearance of antioxidant enzymes.

Additionally, we also observed the decreased levels of autophagy-related proteins in the NAC-treated group, which further demonstrated that ROS could initiated autophagy in DB-treated group.

## Conclusions

In summary, our findings confirmed that DB can cause hepatocellular injury, which is consistent with clinical research. These results suggested that DB-induced autophagy was regulated by ROS and functioned as a self-protective mechanism to prevent cellular further injury in cells. However, excessive autophagy can result in cellular damage, as evidenced by ROS-induced mitochondrial dysfunction following DB treatment. These observations demonstrated that mitochondria are the target organelles for DB-induced hepatotoxicity, and that clearance of oxidative free radicals is likely an ideal therapeutic approach.

## Author Contributions

JY and MX designed and performed most of the experiments, and contributed equally to this work. JY wrote the manuscript and YL did the revision. SZ, YL, XL, DC, and JR assisted in completing part of the experiments. LZ was the principal investigator of this work and was involved in the design of this trial. All the authors approved the final version of the manuscript and agreed to be accountable for the content of the work.

## Conflict of Interest Statement

The authors declare that the research was conducted in the absence of any commercial or financial relationships that could be construed as a potential conflict of interest.
