# Supplementary material for: Diosbulbin B-Induced Mitochondria-Dependent Apoptosis in L-02 Hepatocytes is Regulated by Reactive Oxygen Species-Mediated Autophagy
Source: Front Pharmacol. 2019 Jun 19;10:676. doi: 10.3389/fphar.2019.00676 (PMC6593359; doi:10.3389/fphar.2019.00676)
Supplement: Supplementary file 1 [file DataSheet_1.pdf]

Supplementary Material

Diosbulbin B Induced Mitochondria-Dependent Apoptosis in L-02 Hepatocytes is Regulated by ROS-Mediated Autophagy

Jing Ye[1a], Mei Xue[2a] Yamin Liu[3], Sirui Zhu[1], Yu Li[1], Xiaoli Liu[1], Danhong Cai[1], Jia Rui, Liang Zhang[1b]

[1]College of Pharmacy, Nanjing University of Chinese Medicine, Nanjing, Jiangsu, China
[2]College of Preclinical Medicine, Nanjing University of Chinese Medicine, Nanjing, Jiangsu, China

[3]Department of Pharmacy, Zhongda Hospital, Southeast University, Nanjing Jiangsu, China

[a]These authors contributed equally to this work.

[b]Address correspondence to Liang Zhang, College of Pharmacy, Nanjing University of Chinese Medicine, Nanjing, Jiangsu, China (Zhangl_1999@163.com)

Supplementary Fig S1

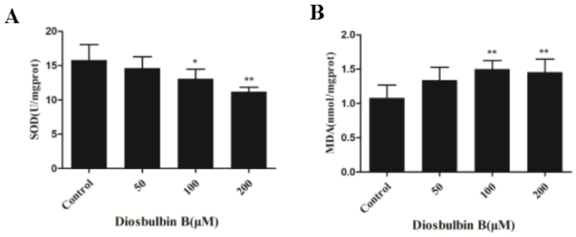

(A-B) The levels of MDA and SOD in cells treated with DB(0,50,100,200μM) for 48h were analyzed with a microplate reader. **P<0.01, *P<0.05,compared to control group.

Supplementary Fig S2

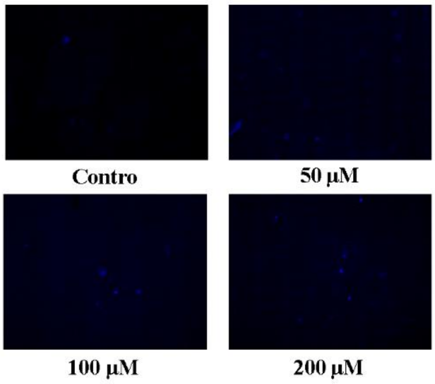

MDC staining assay was applied to detect autophagy in L-02 cells treated with DB(0,50,100,200µM) for 48h.

Supplementary Fig S3

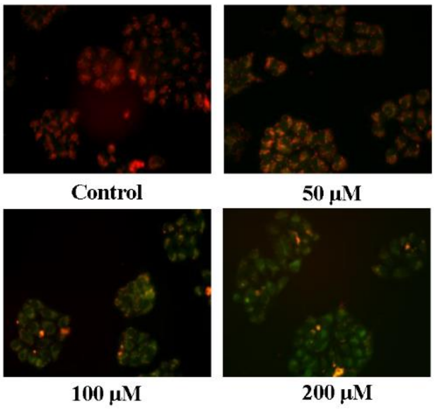

The fluorescent images of JC-1 staining in L-02 cells treated with DB(0,50,100,200µM) for 48h

Supplementary Fig S4

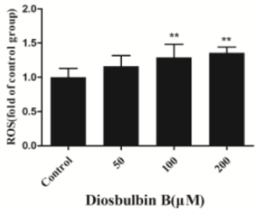

The fluorescence intensity of ROS in L-02 cells treated with DB(0,50,100,200µM) for 48h

Supplementary Fig S5

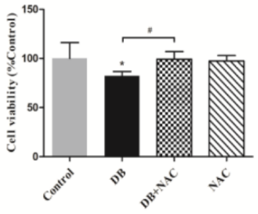

Cell viability of L-02 cells applied with medium (control group), DB(100μM) and NAC(5mM) or the combination. $^{*}P<0.05$,compared to control group.
